# Efficacy of adjunctive antidepressants in treating negative symptoms of schizophrenia: a systematic review and network meta-analysis

**DOI:** 10.1017/S0033291725101803

**Published:** 2025-10-24

**Authors:** Yuting Li, Minglan Yu, He Yang, Lu Shi, Tingting Wang, Rongfang He, Kezhi Liu, Wei Dong, Xuemei Liang, Bo Xiang

**Affiliations:** 1 Department of Psychiatry, Fundamental and Clinical Research on Mental Disorders Key Laboratory of Luzhou, Medical Laboratory Center, Laboratory of Neurological Diseases & Brain Function, Affiliated Hospital of Southwest Medical University, Luzhou, China; 2 Institute of Cardiovascular Research, Southwest Medical University, Luzhou, China; 3 Zigong Mental Health Center, Zigong Affiliated Hospital of Southwest Medical University, Zigong, China; 4 Zigong Institute of Brain Science, Zigong, China

**Keywords:** antidepressive agents, negative symptoms, network meta-analysis, schizophrenia, treatment, psychopharmacology, psychosis, antidepressives, systematic review

## Abstract

**Background:**

The treatment response for the negative symptoms of schizophrenia is not ideal, and the efficacy of antidepressant treatment remains a matter of considerable controversy. This systematic review and meta-analysis aimed to assess the efficacy of adjunctive antidepressant treatment for negative symptoms of schizophrenia under strict inclusion criteria.

**Methods:**

A systematic literature search (PubMed/Web of Science) was conducted to identify randomized, double-blind, effect-focused trials comparing adjuvant antidepressants with placebo for the treatment of negative symptoms of schizophrenia from database establishment to April 16, 2025. Negative symptoms were examined as the primary outcome. Data were extracted from published research reports, and the overall effect size was calculated using standardized mean differences (SMD).

**Results:**

A total of 15 articles, involving 655 patients, were included in this review. Mirtazapine (*N* = 2, *n* = 48, SMD −1.73, CI −2.60, −0.87) and duloxetine (*N* = 1, *n* = 64, SMD −1.19, CI −2.17, −0.21) showed significantly better efficacy for negative symptoms compared to placebo. In direct comparisons between antidepressants, mirtazapine showed significant differences compared to reboxetine, escitalopram, and bupropion, but there were no significant differences between other antidepressants or between antidepressants and placebo. No publication bias for the prevalence of this condition was observed.

**Conclusions:**

These findings suggest that adjunctive use of mirtazapine and duloxetine can effectively improve the negative symptoms of schizophrenia in patients who are stably receiving antipsychotic treatment. Therefore, incorporating antidepressants into future treatment plans for negative symptoms of schizophrenia is a promising strategy that warrants further exploration.

## Introduction

Schizophrenia is a heterogeneous clinical syndrome that includes many domains of psychopathology, with patients varying in their pathological manifestations (Buchanan & Carpenter, 1994). People with schizophrenia experience ‘positive’ psychotic symptoms, including delusions, hallucinations, and disorganized thoughts and behavior, as required by diagnostic criteria. Research on the clinical manifestations of negative symptoms indicates that there are at least five symptoms, including blunted affect, alogia, avolition, social withdrawal, and anhedonia. Factor analysis suggests that these characteristics can be divided into two subdimensions: reduced expression and avolition/apatheia (Blanchard & Cohen, 2006; Correll & Schooler, 2020; Foussias et al., 2014; Kirkpatrick et al., 2006, 2011; Stiekema et al., 2016). The presence of negative symptoms and cognitive impairment is frequently observed in schizophrenia and is known to be a significant contributor to impaired functional abilities (Villalta-Gil et al., 2006). Negative symptoms are the primary etiological factor contributing to compromised occupational (Velligan et al., 2009; Ventura et al., 2009) and social functioning (Lin et al., 2013), as well as imposing a significant burden on caregivers of individuals affected (Magliano et al., 1998, 2000). Consequently, negative symptoms play a pivotal role in the considerable financial implications of schizophrenia for healthcare systems and society (McEvoy, 2007) and have been recognized as an unaddressed therapeutic requirement (Kirkpatrick et al., 2006).

Despite considerable improvements in our understanding of schizophrenia and its multifaceted symptoms involving thinking, perception, and affect, drugs that effectively treat all of these aspects are still lacking (Kane & Correll, 2010; Maric et al., 2016). Most of the currently approved antipsychotic medications exert their effects through dopamine regulation (Carlsson & Carlsson, 2006), primarily used to treat positive symptoms, agitation, and hostility. However, important functional domains of negative symptoms and cognitive dysfunction remain relatively unaddressed (Carbon & Correll, 2014). This fact has stimulated research beyond dopamine neurotransmitter regulation, aiming to address not only positive symptoms but also other areas of schizophrenia (Köster, Carbon, & Correll, 2014).

The treatment of negative symptoms has also evolved from a single antipsychotic drug to a combination of other treatments, such as physical intervention (Fitzgerald & Daskalakis, 2008; Freitas et al., 2009), psychological interventions (Mössler et al., 2011; Sarin et al., 2011; Wykes et al., 2008), anti-Parkinson drugs (Mössler et al., 2011; Paraschakis, 2014; Rezaei et al., 2013), anti-epileptic drugs (Nakamura et al., 2009), and non-pharmacological treatment. The most common combination is the combination of antipsychotics and antidepressants (Mao & Zhang, 2015). A meta-analysis found that, in Western countries, approximately 30% (range: 11–40%) of schizophrenia patients take antidepressants (Mao & Zhang, 2015). In clinical care, antidepressants are increasingly used in patients with schizophrenia, most commonly to target negative, cognitive, and depressive symptoms. The potential therapeutic role of antidepressants in schizophrenia is supported by studies (Cornblatt et al., 2007; Fusar-Poli et al., 2015) that suggest that antidepressants can reduce the transition from a high risk of clinical psychosis to presenting psychosis, and that they can reduce psychosis relapse in patients with schizophrenia and postpsychotic depression (Siris et al., 1994).

Antidepressants are primarily classified into categories such as monoamine oxidase inhibitors, 5-hydroxytryptamine (5-HT) and norepinephrine (NE) reuptake blockers, selective serotonin reuptake inhibitors, serotonin antagonists and reuptake inhibitors, serotonin modulators and agonists, and NE and dopamine reuptake inhibitors. The diverse mechanisms of these drugs suggest their potential in treating schizophrenia’s negative symptoms. Past research has suggested that antidepressants may influence schizophrenia’s negative symptoms through their effects on γ-aminobutyric acid (GABA) and glutamate neurotransmitters (Pehrson et al., 2016), as well as their action on multiple neurotransmitters like dopamine. A key characteristic of schizophrenia is the altered activity of the dopamine system in different brain regions, which gives rise to various symptom profiles. Antidepressants also target post-synaptic 5-hydroxytryptamine type 3 (5-HT3) receptors and presynaptic α2-adrenergic receptors and indirectly stimulate post-synaptic 5-hydroxytryptamine (serotonin) receptor 1A (5-HT1A) receptors (de Boer, 1996), all of which are implicated in the pathophysiology of schizophrenia. Preceding evidence suggests that 5-HT3 receptor antagonists hold promise for treating schizophrenia due to their potential therapeutic benefits (Costall & Naylor, 1992).

Previous meta-analyses have indicated that the combination of antipsychotic and antidepressant treatment may be effective for the negative symptoms of schizophrenia (Galling et al., 2018; Rummel et al., 2006). The driving factor for overall symptom improvement is the negative symptoms, rather than the reduction of positive symptoms or total symptom severity. It was also found that antidepressants have demonstrated an advantage in improving negative symptoms in studies involving the addition of first-generation antipsychotics, but this was not confirmed in studies involving the addition of second-generation antipsychotics.

However, so far, the efficacy of antidepressants in the treatment of negative symptoms of schizophrenia is still controversial. Although most studies have shown that antidepressants are effective in the treatment of negative symptoms of schizophrenia, several studies have shown that antidepressants cannot improve negative symptoms of schizophrenia (Sepehry et al., 2007; Singh et al., 2010; Usall et al., 2014). These contradictory results may be accounted for by methodological limitations: small sample, short trial length, a range of concomitant antipsychotic regimens, or lack of exclusion of subjects with depressive symptoms. Given the current scenario, the effectiveness of antipsychotic medications in addressing the negative symptoms of schizophrenia is restricted, prompting a shift toward combined therapies, where the use of adjunctive antidepressants emerges as a beneficial strategy.

In this meta-analysis, we conducted a comprehensive review of recent studies on the adjunctive use of antidepressants in treating negative symptoms of schizophrenia. Previous meta-analyses did not adequately focus on negative symptoms in the inclusion criteria, which may lead to bias in the research results. However, in this study, we specifically focused on negative symptoms and made them a key aspect of the inclusion criteria. The improvement in the inclusion criteria lies in our clear emphasis on the assessment of schizophrenia’s negative symptoms and prioritizing their evaluation. We also ensured that the scales used to assess negative symptoms are flexible and can accommodate different measurement methods. By incorporating this focus, we aim to provide more detailed and targeted insights into the effects of interventions on negative symptoms, which are often underrepresented in schizophrenia research. At the same time, because there are common features between negative symptoms and depressive symptoms, it is necessary to focus on depressive symptoms, as detailed in Supplementary Table S1. Consequently, we systematically reviewed the effects of antidepressant use in patients with negative symptoms and incorporated it as an essential criterion for selection. Our objective is to determine which antidepressant exhibits superior efficacy compared to placebo while also exploring potential differences among various antidepressants and to provide valuable insights for future therapeutic approaches targeting negative symptoms in individuals with schizophrenia.

## Methods

### Search and selection criteria

In this systematic review and meta-analysis, we thoroughly examined the reference lists of previous systematic reviews and meta-analyses pertaining to this topic within our research group, while also conducting an extensive search. Our literature search encompassed Web of Science (from its inception until April 16, 2025) and PubMed (from its establishment until April 16, 2025). We conducted a comprehensive search employing various combinations of keywords pertaining to the three central themes: negative symptoms, schizophrenia, and antidepressants. The specific search terms and search strategy can be found in the Supplementary Materials.

Inclusion criteria for the study: (1) patients aged 18 to 65 years; (2) clearly diagnosed with schizophrenia, regardless of gender, race, or diagnostic criteria used; (3) the inclusion criteria or primary outcome measure specifically focus on the negative symptoms of schizophrenia, without restriction on the scales used to assess negative symptoms; (4) the inclusion criteria focus on depressive symptoms (regardless of the scale used) and exclusions were made; (5) randomized controlled trials, including studies on all existing antidepressants and placebo treatments on the market; (6) written in English. exclusion criteria: (1) studies involving comorbid psychiatric disorders and substance abuse; (2) abstracts, reviews, case reports, commentaries, letters, and protocols; (3) studies from which valid data cannot be obtained after contacting the corresponding author and foreign experts for literature searches. The primary outcome measure focused on assessing changes in negative symptoms of schizophrenia using either the scale for Assessment of Negative Symptoms (SANS; Andreasen, 1982) or the scale for Positive and Negative Symptoms-Negative Subscale (PANSS-N; Kay et al., 1987, 1988). These scales have demonstrated high internal consistency and external validity among various population groups; moreover, they are considered relatively comparable (Czobor et al., 1991; McAdams et al., 1996; Thiemann et al., 1987).

### Outcome measures and data extraction

The primary outcome was the mean change from baseline to endpoint in negative symptoms of schizophrenia as measured by negative subscales of the PANSS-N (Fusar-Poli et al., 2015; Kay et al., 1987) or the SANS (Andreasen, 1989). If the change data were not available, we used the mean score at the study endpoint of these scales. The articles’ quality was classified according to the risk of bias tool (Cumpston et al., 2019). The selected literature was assessed based on seven indicators: random sequence generation, allocation concealment, blinding of investigators and subjects, blinded evaluation of study outcomes, completeness of outcome data, selective reporting of study outcomes, and other sources of bias. If all criteria were met with low risk factors present or accounted for appropriately in case there were no high-risk indicators identified, then it was determined that the study had a low risk of bias. In case one or more criteria remained unclear without any high-risk indicators being found, then it was considered that the study had an unclear risk of bias. However, if one or more indicators were deemed as high risk, then it was concluded that the study had a high risk of bias. Two resident psychiatrists from the department of psychiatry independently reviewed the results of the updated search, obtained full-text articles, assessed eligibility criteria, and extracted data. The data analysis was performed by one of the psychiatrists, who then verified and checked the results with another psychiatrist. If an article meets the inclusion criteria but complete data cannot be extracted through various methods, such as contacting the corresponding author or inviting foreign experts to conduct literature searches, the article will be excluded in order to minimize the result bias caused by missing data. Any discrepancies were resolved through discussion.

### Statistical analysis

This network meta-analysis (NMA) focuses on the measurement methods for the reduction of negative symptom scores. Considering the different scales used in the included studies to measure the reduction of negative symptoms, the overall effect size for the primary outcome measure was calculated using the standardized mean difference (SMD). The meta-analysis was conducted by combining the standardized effect sizes between trials using a random-effects model, which accounted for the heterogeneity between studies due to variability in case combinations and trial settings. We performed a sensitivity analysis, and the results showed that no study had an exceptionally large impact on the pooled effect size, indicating that our effect size remained stable. Global and local inconsistency analyses were performed using Stata MP 17 (64-bit edition) to assess the viability of integrating direct and indirect comparisons across different scales. Heterogeneity was assessed utilizing the chi-square test, *I* ^2^ statistic, and forest plots, with I2 values ranging from 0% to 100% (where smaller values indicate lower heterogeneity). A *p*-value below 0.05 denoted a significant reduction in heterogeneity. The NMA encompassed the creation of network diagrams, pairwise comparison forest plots, funnel plots, and cumulative ranking curves. The network diagram depicts the interrelationships among various intervention measures, where each node symbolizes a distinct intervention, and each edge signifies a direct comparison between two interventions. Moreover, the size of a node reflects the frequency of the corresponding drug as a comparator in the study. The thickness of edges connecting two nodes is directly related to the number of studies that include the two directly compared intervention measures. The funnel plot and Egger’s test were used to assess publication bias. Surface under the cumulative ranking curve (SUCRA) and average ranking were employed to evaluate each outcome. The greater the area under the curve for each drug, the higher the likelihood that it signifies the optimal intervention.

## Results

### Search results

**Figure 1.**
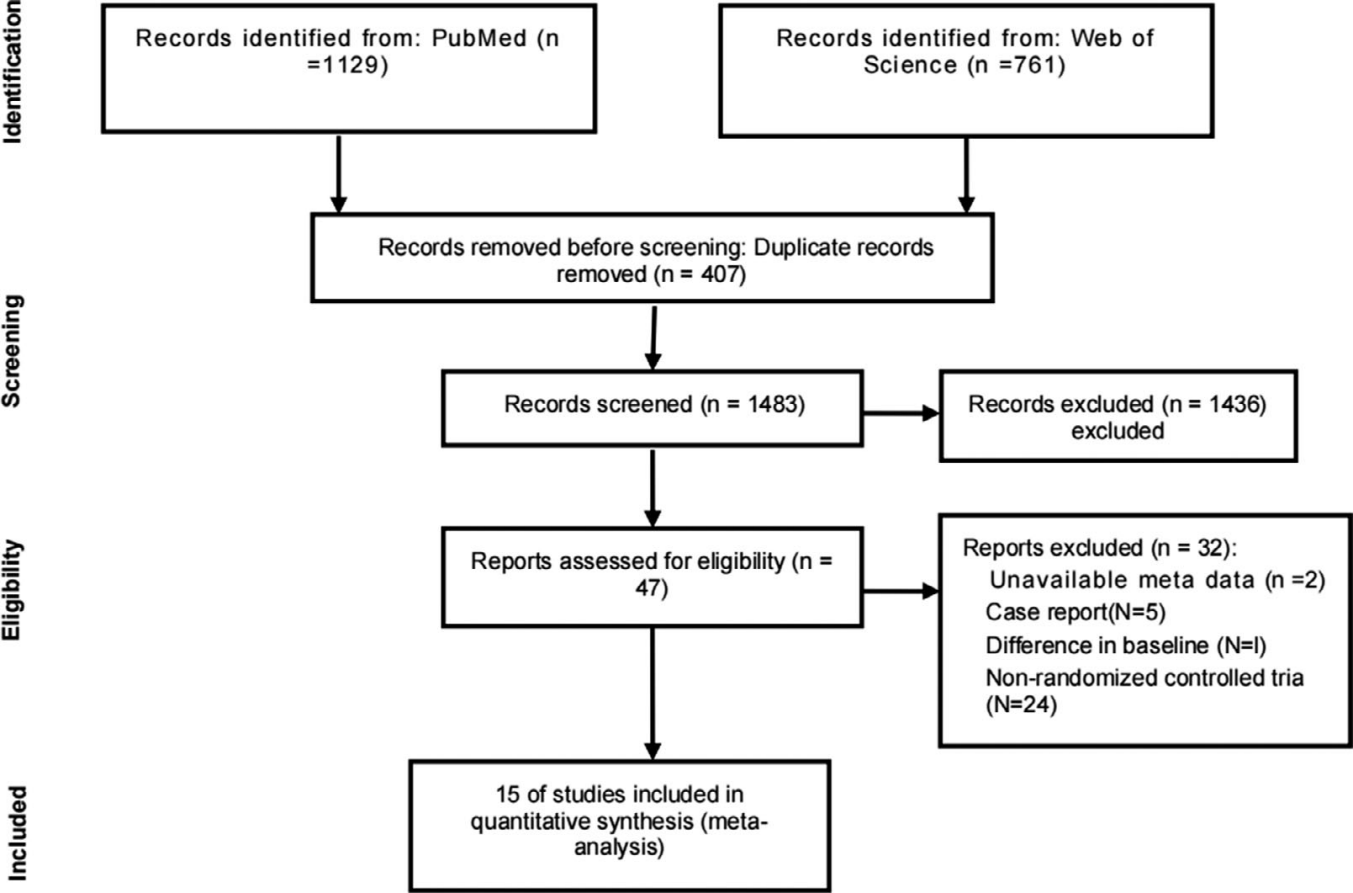
Flow chart of the systematic literature search and excluded and included studies.

The literature search initially yielded 1890 potentially relevant studies. After removing duplicates, 1483 studies remained. Of these 1483 studies, 1436 were excluded after title and abstract screening, leaving 47 studies for further full-text review (reasons for exclusion are shown in Figure 1). Finally, 15 articles (*n* = 655) met the inclusion criteria and were included in this systematic review and meta-analysis.

### Characteristics of the included studies

Table 1 lists the detailed information of all included studies. Among 481 patients with gender specified, 312 were male (65%), and the median trial duration was 8 weeks (range: 4–48 weeks). The risk of bias assessment is shown in Supplementary Figure S1. Several trials lacked clear details on the randomization procedure and allocation concealment. In about 40% of the studies, the blinding of patients and staff remained unclear. However, there was no indication of high risk related to insufficient blinding. The risk of bias in outcome assessment related to blinding showed similar uncertainty (40%), but did not suggest any high risk.

**Table 1.** Included studies, patients, and treatment characteristics

Study	Design	n	Duration (weeks)	Setting	Study tools	Inclusion criteria: depressive symptoms negative symptoms	Symptom severity bl	Changes in the severity of symptoms	Mean age	Male (%)	Illness duration	Antipsychotic: mean dose	Antidepressant: Ø dose
Mozen-Zadeh 2020	DB/RCT	68	8	Inpatients	Negative symptoms (PANSS-N)	Exclusion of MDD Negative symptoms required: PANSS-N>16	Control group: PANSS-N = 19.2 ± 3.2 Intervention group: PANSS-N = 19.4 ± 3.4	Control group: PANSS-N = –0.6 ± 1.4 Intervention group: PANSS-N = −2.4 ± 2.2	Control group: 32.9 Intervention group: 34.4	69.1	Control group: 8.7 Intervention group: 9.4	RIS: 6	VORT (*n* = 34): 20 mg/day
Silver 2000	DB/RCT	46	6	Inpatients	Negative symptoms (SANS)	Exclusion of MDD Persistent negative symptoms required: SANS ≥ moderate illness severity	Control group: SANS = 71.3 ± 16.8 Intervention group: SANS = 74.0 ± 14.1	Control group: SANS = 3.7 ± 18.2 Intervention group: SANS = –4.4 ± 17.1	43	41.3	19.7	FGA: mixed (NR)	FLU (*n* = 30): 50–100 mg/day
Jockers-Scherübl 2005	DB/RCT	25	12	Outpatients	Negative symptoms (PANSS-N)	Exclusion of MDD or HAM-D>12 Negative symptoms required: PANSS-N>20	Control group: PANSS-N = 26.9 ± 4.3 Intervention group: PANSS-N = 32.3 ± 4.4	Control group: PANSS-N = –4.3 ± 6.0 Intervention group: PANSS-N = –9.2 ± 5.0	Control group: 40.8 Intervention group: 40.0	48.0	9.9	AP: mixed (NR)	PAR (*n* = 11): 20–30 mg/day
Zoccali 2004	DB/RCT	20	8	Outpatients	Negative symptoms (SANS)	Exclusion of MDD or HAM-D>20 Negative symptoms required: one of the five global SANS subscales ≥3	Control group: SANS = 51 ± 5.3 Intervention group: SANS = 48.9 ± 6.9	Control group: SANS = 0.0 ± 6.7 Intervention group: SANS = –12.8 ± 6.1	Control group: 33.4 Intervention group: 30.7	65.0	Control group: 13.6 Intervention group: 9.6	CLO: 320/325	MIR (*n* = 10): 30 mg/day
Ding 2018	DB/RCT	54	8	Inpatients	Negative symptoms (PANSS-N)	Depressive symptoms irrelevant Negative symptoms required: PANSS-N score ≥ 4 in at least three items or ≥ 5 in at least two items	Control group: PANSS-N = 32.5 ± 5.6 Intervention group: PANSS-N = 32.1 ± 4.9	Control group: PANSS-N = –1.7 ± 4.1 Intervention group: PANSS-N = –4.3 ± 4.1	Control group: 49.7 Intervention group: 42.4	50.0	Control group: 22.8 Intervention group: 18.8	AP: mixed (NR)	ESC (*n* = 28): 5–20 mg/day
Barnes 2016	DB/RCT	42	48	Inpatients	Negative Symptoms (PANSS-N)	Exclusion of MDD Persistent negative symptoms required: PANSS-N ≥ 20 with at least three of the seven items on the negative symptom subscale rated ≥3.	Control group: PANSS-N = 25.7 ± 5.3 intervention group: PANSS-N = 25.3 ± 3.9	Control group: PANSS-N = –4.1 ± 5.7 intervention group: PANSS-N = –5.1 ± 4.6	Control group: 45.1 intervention group: 43.0	NR	NR	AP: mixed (NR)	CIT (*n* = 19): 20–40 mg/day
Shafti 2015	DB/RCT	50	12	Inpatients	Negative symptoms (SANS)	Exclusion of MDD or HAM-D >10 Persistent negative symptoms required: High negative symptom scores (more than 55% of total SANS, ≥66), low positive symptom scores (less than 55% of total SAPS, ≤96)	Control group: SANS = 80.4 ± 2.5 intervention group: SANS = 79.9 ± 1.2	Control group: SANS = –1.3 ± 5.1 intervention group: SANS = –5.7 ± 3.6	NR	100.0	NR	HAL: 5–20	REB (*n* = 25): 4 mg/day
Usall 2014	DB/RCT	64	24	NR	Negative symptoms (PANSS-N) /Negative symptoms (SANS)	Exclusion of MDD or HAM-D >20 Persistent negative symptoms required: 1 or more negative symptom with a severity score greater than 4 on the PANSS negative scale.	Control group: PANSS-N = 26.2 ± 6.4/SANS = 62.3 ± 19.2 intervention group(Reb): PANSS-N = 25.5 ± 6.77/SANS = 61.6 ± 19.7 intervention group(Cit): PANSS-N = 25.9 ± 5.2/SANS = 61.2 ± 17.9	Control group: PANSS-N = –3.6 ± 6.0 intervention group(Reb): PANSS-N = –5.8 ± 7.7 intervention group(Cit): PANSS-N = –6.09 ± 5.07	Control group: 44.2 intervention group (Reb): 40.0 intervention group(Cit): 42.5	NR	NR	OLA/RIS: NR	CIT (*n* = 17): 15–30 mg/day REB (*n* = 24): 4–8 mg/day
Hinkelmann 2013	TB/RCT	51	4	NR	Negative Symptoms (PANSS-N)	Depressive symptoms irrelevant Negative symptoms required: scoring ≥4 points in at least 1 item of the PANSS-N	Control group: PANSS-N = 26.7 ± 7.9 intervention group(Reb): PANSS-N = 25.6 ± 6.4 intervention group (Cit): PANSS-N = 24.7 ± 10.2	Control group: PANSS-N = –7.6 ± 7.0 intervention group(Reb): PANSS-N = –4.1 ± 7.7 intervention group(Cit): PANSS-N = –5.60 ± 9.6	Control group: 38.3 intervention group(Reb): 42.1 intervention group(Cit): 38.5	66.7	Control group: 9.7 intervention group(Reb): 15.1 intervention group(Cit): 9.2	AP: mixed(NR)	CIT (*n* = 17): 20–40 mg/day REB (*n* = 24): 4–8 mg/day
Abbasi 2010	TB/RCT	38	8	Inpatients	Negative Symptoms (PANSS-N)	Exclusion of MDD or The PANSS depression item score (exclusion level ≥ 4) Persistent negative symptoms required: PANSS-N ≥ 15	Control group: PANSS-N = 27.0 ± 4.8 intervention group: PANSS-N = 27.7 ± 4.5	Control group: PANSS-N = –7.65 ± 2.90 intervention group: PANSS-N = –13.45 ± 4.04	Control group: 34.5 intervention group: 33.4	NR	Control group: 7.4 intervention group: 7.8	RIS: 2–6	MIR (*n* = 19): 15–30 mg/day
Buchanan 1996	DB/RCT	33	8	Outpatients	Negative symptoms (SANS)	Depressive symptoms irrelevant Negative symptoms required: a score of at least 2 on one of the five global scales of the SANS or SANS≥20	Control group: SANS = 23.9 ± 10.9 intervention group: SANS = 29.5 ± 15.3	Control group: SANS = 2.0 ± 11.4 intervention group: SANS = –0.6 ± 15.2	Control group: 32.8 intervention group: 36.8	69.7	Control group: 15.6 intervention group: 16.2	CLO: 511.7/457.4	FLU (*n* = 14): 20–80 mg/day
Spina 1994	DB/RCT	30	12	Inpatients	Negative symptoms (SANS)	Exclusion of MDD or HAM-D >20 Negative symptoms required: a score of at least ‘moderate’ on one of the five global scales of the SANS	Control group: SANS = 62.5 ± 11.3 intervention group: SANS = 63.4 ± 8.1	Control group: SANS = –3.2 ± 11.2 intervention group: SANS = –21.4 ± 13.3	Control group: 45.4 intervention group: 46.3	NR	NR	AP(NR)	FLU (*n* = 14): 20 mg/day
Silver 1992	DB/RCT	30	7	Inpatients	Negative symptoms (SANS)	Depressive symptoms irrelevant Negative symptoms required: a score of at least ‘moderate’ on one of the five global scales of the SANS	Control group: SANS = 52.9 ± 14.6 intervention group: SANS = 57.8 ± 14.5	Control group: SANS = –13.8 ± 13.1 intervention group: SANS = –28.9 ± 13.5	Control group: 42 intervention group: 41	63.3	NR	AP(NR)	FLU (*n* = 15): 50–100 mg/day
Nikbakhat 2016	TB/RCT	64	8	Inpatients	Negative Symptoms (PANSS-N)	Exclusion of MDD or HDRS ≥14 or a score of ≥4 on a PANSS depression item Persistent negative symptoms required: Unclear/Unspecific	Control group: PANSS-N = 17.4 ± 1.4 intervention group: PANSS-N = 17.5 ± 1.9	Control group: PANSS-N = –4.2 ± 1.1 intervention group: PANSS-N = –6.2 ± 2.1	Control group: 34.2 intervention group: 33.9	67.2	Control group: 8.9 intervention group: 9.0	RIS: 4–6	DUI (*n* = 32): 60 mg/day
Yassini 2014	DB/RCT	40	12	Inpatients	Negative symptoms (SANS)	Exclusion of MDD Persistent negative symptoms required: Unclear/Unspecific	Control group: SANS = 96.1 ± 14.3 intervention group: SANS = 100.1 ± 11.3	Control group: SANS = –0.5 ± 14.0 intervention group: SANS = 0.6 ± 11.8	Control group: 49.0 intervention group: 49.6	62.5	Control group: 23.7 intervention group: 22.3	AP(NR)	BUP (*n* = 20): 75–100 mg/day

*Note:* AD, antidepressant; AP, antipsychotic; bl, baseline; BPRS, brief psychiatric rating scale; BPRS-P, BPRS positive subscale; BUP, Bupropion; CIT, citalopram; CLO, clozapine; d, days; DB/RCT, Double-blind, randomized, placebo-controlled trial; DUI, duloxetine; ESC, escitalopram; FGA, first-generation antipsychotic; FLU, fluoxetine; FLV, fluvoxamine; HAL, haloperidol; HDRS, Hamilton depression rating scale; MDD, major depressive disorder; MIR, mirtazapine; NR, not reported; OLZ, olanzapine; PANSS, positive and negative syndrome scale; PANSS-N, PANSS negative subscale; PAR, paroxetine; REB, reboxetine; RIS, risperidone; SANS, scale for the assessment of negative symptoms.

**Figure 2.**
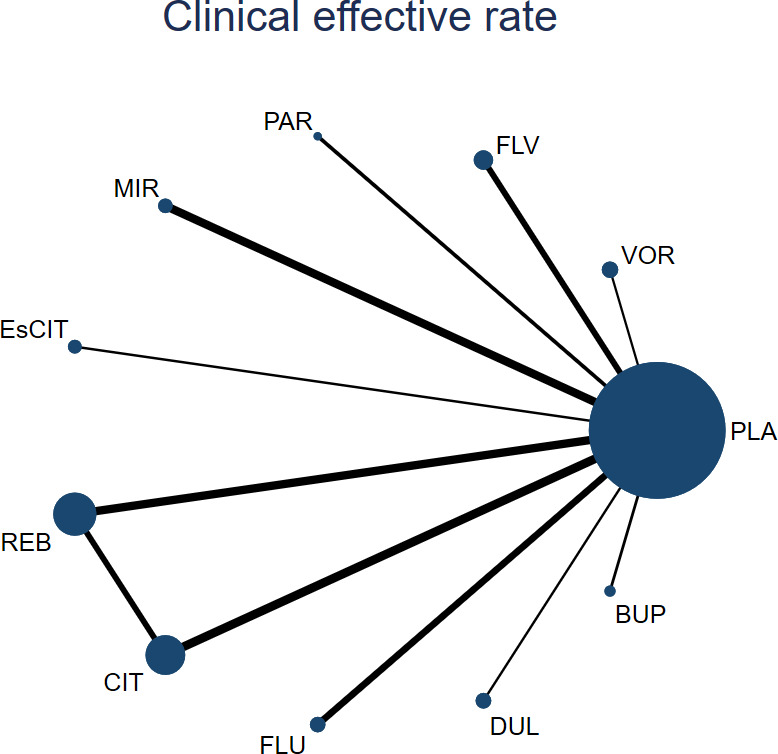
Network geometry. Among them, PLA represents placebo, VOR represents vortioxetine, FLV represents fluvoxamine, PAR represents paroxetine, MIR represents mirtazapine, EsCIT represents escitalopram, REB represents reboxetine, CIT represents citalopram, FLU represents fluoxetine, DUL represents duloxetine, and BUP represents bupropion. The figure shows the results of a network meta-analysis that directly compares the 10 interventions. The width of the lines is proportional to the number of lines comparing each pair of interventions, and the size of each blue dot is proportional to the sample size of the interventions.

Figure 2 shows the qualified comparison network for the main outcomes. The identified studies included drugs vortioxetine, fluvoxamine, paroxetine, mirtazapine, escitalopram, reboxetine, citalopram, fluoxetine, duloxetine, bupropion, placebo. Fifteen of the included studies met our criteria for patients with negative symptoms and included treatments: vortioxetine (*N* = 1), fluvoxamine (*N* = 2), paroxetine (*N* = 1), mirtazapine (*N* = 2), escitalopram (*N* = 1), reboxetine (*N* = 3), citalopram (*N* = 3), fluoxetine (*N* = 2), duloxetine (*N* = 1), bupropion (*N* = 1). Figure 2 shows the relationships between 10 different interventions, with each blue dot representing an intervention and each black line representing a direct comparison between two interventions. Based on Figure 2, the blue dot for the reboxetine group has the largest area. Therefore, in the included studies, the reboxetine group was the most commonly compared group. Based on the width of the black lines connecting the two blue dots, which represent the proportion of studies comparing the two interventions directly, the black line between reboxetine and placebo is the thickest, indicating that the number of studies comparing reboxetine and placebo directly was the highest. Similarly, this also occurs in the direct comparison of citalopram and placebo.

### Efficacy of treatments for negative symptoms

Ten drugs were compared with a placebo (Table 2). For mirtazapine, two placebo-controlled trials were conducted. Mirtazapine significantly outperformed placebo in treating negative symptoms (*N* = 2, *n* = 48, SMD −1.73, CI −2.60, −0.87). Based on one trial, duloxetine significantly outperformed placebo in negative symptoms (*N* = 1, *n* = 64, SMD −1.19, CI −2.17, −0.21). In direct comparisons between antidepressants, significant differences were found between mirtazapine and reboxetine, citalopram, and bupropion, while no significant differences were observed in direct comparisons between other antidepressants or between antidepressants and placebo. We also made predictions regarding the efficacy of antidepressants, with the area under the curve representing the efficacy ranking of antidepressants in treating negative symptoms, as shown in Supplementary Figure S2. It can be seen that mirtazapine (MIR) has the largest area under the curve, followed by duloxetine (DUL). The area under the curve for bupropion (BUP) is nearly identical to that of placebo (PLA). This indicates that mirtazapine and duloxetine are more effective in treating negative symptoms of schizophrenia compared to other antidepressants, while bupropion is less effective than other antidepressants. Table 3 shows the cumulative probability prediction values for the ranking of each drug, with a higher probability indicating better drug efficacy. The probability of mirtazapine being effective is the highest at 93.9%, followed by duloxetine at 76.2%.

**Table 2.** Results of network meta-analysis of the improvement rate of negative symptoms of schizophrenia (SMD value and 95% CI)

MIR										
−0.54 (−1.85, 0.77)	DUL									
−0.77 (−2.06, 0.52)	−0.23 (−1.61, 1.14)	VOR								
−0.89 (−2.34, 0.56)	−0.35 (−1.87, 1.18)	−0.12 (−1.63, 1.40)	PAR							
−0.96 (−2.13, 0.20)	−0.42 (−1.68, 0.83)	−0.19 (−1.44, 1.05)	−0.08 (−1.49, 1.33)	FLU						
−0.99 (−2.14, 0.15)	−0.45 (−1.69, 0.78)	−0.22 (−1.45, 1.00)	−0.11 (−1.50, 1.28)	−0.03 (−1.12, 1.06)	FLV					
−1.11 (−2.42, 0.20)	−0.57 (−1.96, 0.82)	−0.34 (−1.72, 1.04)	−0.22 (−1.75, 1.31)	−0.15 (−1.41, 1.12)	−0.12 (−1.36, 1.13)	EsCIT				
−1.42 (−2.46, −0.38)	−0.88 (−2.02, 0.26)	−0.65 (−1.77, 0.47)	−0.53 (−1.83, 0.77)	−0.46 (−1.43, 0.52)	−0.43 (−1.37, 0.52)	−0.31 (−1.45, 0.83)	REB			
−1.50 (−2.54, −0.45)	−0.96 (−2.10, 0.19)	−0.73 (−1.85, 0.40)	−0.61 (−1.92, 0.70)	−0.53 (−1.52, 0.45)	−0.50 (−1.46, 0.45)	−0.39 (−1.54, 0.76)	−0.08 (−0.73, 0.58)	CIT		
−1.81 (−3.16, −0.47)	−1.27 (−2.69, 0.15)	−1.04 (−2.45, 0.37)	−0.93 (−2.48, 0.63)	−0.85 (−2.15, 0.45)	−0.82 (−2.09, 0.46)	−0.70 (−2.13, 0.72)	−0.39 (−1.57, 0.79)	−0.32 (−1.50, 0.87)	BUP	
−1.73 (−2.60, −0.87)	−1.19 (−2.17, −0.21)	−0.96 (−1.93, 0.00)	−0.85 (−2.01, 0.32)	−0.77 (−1.56, 0.02)	−0.74 (−1.49, 0.01)	−0.62 (−1.61, 0.36)	−0.31 (−0.89, 0.26)	−0.24 (−0.82, 0.35)	0.08 (−0.95, 1.11)	PLA

*Note:* BUP, bupropion; CIT, citalopram; DUL, duloxetine; EsCIT, escitalopram; FLU, fluoxetine; FLV, fluvoxamine; MIR, mirtazapine; PAR, paroxetine; PLA, placebo; REB, reboxetine: SMD, the standardized mean difference; and VOR, vortioxetine.

**Table 3.** Cumulative probability predicted values for each drug

Treatment	SUCRA	PrBest
PLA	11.9	0.0
VOR	66.8	6.9
FLV	56.4	1.5
PAR	59.9	7.4
MIR	93.9	64.4
EsCIT	49.7	2.2
REB	32.7	0.0
CIT	27.4	0.0
FLU	58.5	2.3
DUL	76.2	15.1
BUP	16.6	0.1

*Note*: The SUCRA represents the area under the curve, indicating better drug efficacy with a larger area. Best probability ranks drug efficacy as a percentage, with higher percentages indicating better efficacy. When there is a discrepancy, the area under the curve result should be used. BUP, bupropion; CIT, citalopram; DUL, duloxetine; EsCIT, escitalopram; FLU, fluoxetine; FLV, fluvoxamine; MIR, mirtazapine; PAR, paroxetine; PLA, placebo; REB, reboxetine; and VOR, vortioxetine.

### Quality evaluation

Supplementary Figure S1 summarizes the results of the quality assessment. All 15 studies were randomized controlled clinical trials (RCTs). The included RCTs were assessed using the Cochrane Collaboration’s tool for assessing the risk of bias. Ten studies had a low risk of bias, and 5 studies had an unclear risk of bias. The quality assessment results of each study were statistically different. All the 15 studies were blinded, of which 12 were double-blind and 3 were triple-blind.

### Publication bias

The funnel plot (Supplementary Figure S3) illustrates that studies with smaller sample sizes are symmetrically arranged at the base of the graph, while those with larger sample sizes are distributed toward the top of the funnel plot and converge toward the center of the graph. The overall symmetrical distribution of the plot suggests that the results of the included studies are relatively consistent, with no significant asymmetry observed, indicating a low risk of publication bias. The funnel plot provides a preliminary assessment of publication bias; however, to further evaluate this, we conducted Egger’s test. The results of Egger’s test, with a *p*-value of 0.342 (*p* > 0.05), suggest a low risk of publication bias. Combining the results from the funnel plot and Egger’s test, it can be concluded that the risk of publication bias in the included studies is low.

### Inconsistency test

Stata software was used to test the inconsistency of statistical results. From the pairwise comparison forest plot in Supplementary Figure S4, we can see that the *p* value is 0.258, which is >0.05, indicating that the heterogeneity test is not significant. A closed-loop structure was formed in the network diagram by direct and indirect comparisons, so we conducted a circular inconsistency test for this study (Chaimani & Salanti, 2015), as shown in Supplementary Figure S5. According to Supplementary Figure S5, the results of the node segmentation method show that there is a direct comparison between reboxetine and citalopram, which forms a closed loop. The 95% confidence interval for the closed loop is (0.00, 1.55) and the IF is 0.76. There was no significant difference between the direct and indirect results. Thus, the inconsistency test in our NMA is not significant, which indicates that our results analysis is reliable.

## Discussion

To the best of our knowledge, this study represented the first NMA examining the efficacy of adjunctive antidepressant medication in treating negative symptoms. The results of this meta-analysis indicated that antidepressant medication is superior to placebo in improving overall symptoms, driven by negative symptoms rather than reducing positive or general symptoms. We concentrated our efforts on randomized placebo-controlled trials, encompassing a total of 344 patients in the treatment group and 311 patients in the placebo group. While previous systematic reviews and meta-analyses have touched upon the supplementary treatment of negative symptoms with antidepressants (Fusar-Poli et al., 2015; Sepehry et al., 2007), they lacked specific and stringent inclusion criteria specific to negative symptoms. We also encourage future research to pay more attention to patients with significant negative symptoms or primary negative symptoms and mild positive symptoms.

In the present meta-analysis, we observed that mirtazapine and duloxetine may be superior to other antidepressants in treating negative symptoms. This may be due to some similarities in their mechanisms of action. Mirtazapine has interesting psychopharmacological characteristics: It antagonizes the 5-HT2 and 5-HT3 receptors, indirectly activates the 5-HT1A receptor, and antagonizes the adrenergic a2 receptor (Croom et al., 2009; Schreiber et al., 2002). Duloxetine selectively inhibits the reuptake of serotonin and NE. Serotonin is a key neurotransmitter in schizophrenia: It is associated with the negative and cognitive symptoms of schizophrenia (Fukuda, 2014). Reduced 5-HT1A receptor binding in the amygdala is related to specific components of the negative symptoms of schizophrenia (Yasuno et al., 2004). Literature reviews also show the role of NE in the pathogenesis of negative symptoms in schizophrenia (Yamamoto & Hornykiewicz, 2004). The effects of mirtazapine and duloxetine on serotonin and NE validate the potential mechanisms for treating the negative symptoms of schizophrenia. Consequently, for the future management of negative symptoms in schizophrenia, it is advisable to commence treatment with antipsychotic medications alone. If the initial response is unsatisfactory, considering potential drug interactions, the addition of mirtazapine or duloxetine may be considered as adjunctive treatment for negative symptoms. In the included RCTs, we observed that the minimum effective dose of mirtazapine was 30 mg/d and for duloxetine, 60 mg/d. At the same time, the gradual improvement of negative symptoms during treatment can be monitored to better assess the efficacy of antidepressant therapy.

In 2010, Singh et al. (2010) conducted a meta-analysis aimed at assessing the efficacy of adjunctive antidepressant medication in treating negative symptoms among patients with schizophrenia. The analysis encompassed 23 trials, involving a total of 819 patients. The findings of this meta-analysis revealed the effectiveness of antidepressant medication in alleviating negative symptoms in schizophrenia. Specifically, fluoxetine, trazodone, and ritanserin demonstrated beneficial effects, whereas mirtazapine, reboxetine, mianserin, fluvoxamine, sertraline, paroxetine, and citalopram did not exhibit any favorable effects. There is a difference in conclusions between our study and the aforementioned meta-analysis, which may be due to the fact that this meta-analysis particularly emphasized the importance of negative symptoms in its inclusion criteria and increased the focus on depressive symptoms. Compared to previous studies, we conducted a more rigorous and detailed screening of the study population and sample. This may have led to differences in sample characteristics and baseline data, which could affect the results. Furthermore, this meta-analysis included the latest studies published in recent years, whereas previous studies may not have covered these newer pieces of evidence. As research evidence accumulates and is updated, the results may change. Additionally, publication bias could also affect the consistency of results across different studies. However, overall, the use of antidepressants is considered effective in treating negative symptoms.

This meta-analysis aligns with the recent study conducted by (Galling et al., 2018), which investigated the adjunctive treatment of schizophrenia using antipsychotic and antidepressant medications. Galling et al. found that mirtazapine demonstrated relatively superior efficacy in addressing negative symptoms when compared to other antidepressant medications, a finding that is in line with the results of this study. It should be noted that this study specifically concentrates on evaluating the efficacy of interventions for negative symptoms (Jockers-Scherübl et al., 2005; Moazen-Zadeh et al., 2020). While our findings align with previous research conducted by Galling et al., neither our study nor theirs was able to distinguish between primary and secondary negative symptoms (e.g. those stemming from anxiety, depression, paranoia, extrapyramidal symptoms, or sedation), nor could we differentiate persistent negative symptoms (‘deficit symptoms’) from transient ones (Carbon & Correll, 2014; Carpenter et al., 1988). However, this does not necessarily imply the clinical significance of these effects. First, it is vital to consider that the observed improvement in negative symptoms in most trials might partially stem from the amelioration of secondary negative symptoms. As a result, these findings may potentially overestimate the actual therapeutic efficacy. Second, even though a slight change in symptom assessment scales may be observed, it may not directly translate into an impact on patients’ functional capacity or quality of life. However, the therapeutic effectiveness could also be influenced by the duration of the study and the stage of patients’ disease progression. In this meta-analysis, the included studies predominantly involved patients with a course of illness concentrated around 10–20 years, indicating generally longer durations of illness. Thus, it was difficult for us to determine the potential changes in the efficacy of antidepressants in the early stages of schizophrenia in this meta-analysis. Since negative symptoms are a chronic aspect of schizophrenia, clinical trials must be sufficiently long to demonstrate that the observed treatment response is not temporary. Moreover, certain specific negative symptoms that need to be addressed depend on interactions with the environment, and it may take months to observe measurable and stable improvements. There is a growing consensus that trials should last approximately 6 months to assess the efficacy of treatments for negative symptoms (Laughren & Levin, 2006). However, to our knowledge, there are few published trials with a follow-up period of 6 months. This may be due to the concern that if patients are randomly assigned to receive placebo adjunctive treatment for such an extended period, their negative symptoms, as well as depressive symptoms that may be confused with negative symptoms, could worsen. This creates significant ethical and feasibility obstacles in the design of trials that include a placebo treatment group. Furthermore, the interpretation of clinical outcomes may be confounded. Loss of clinical stability may lead to an increase in paranoid or other symptoms of schizophrenia, which are behaviorally similar to negative symptoms, such as social withdrawal. Conversely, the emergence of psychotic agitation may be confused with a reduction in negative symptoms (Alphs, 2006). In this meta-analysis, we found that duloxetine and mirtazapine showed relatively better efficacy in the adjunctive treatment of schizophrenia negative symptoms for 8 weeks compared to other antidepressants. However, we cannot assess whether the results of long-term adjunctive treatment for negative symptoms would be stable and measurable. The variability in treatment duration among the trials included in this study limits the generalizability of the findings. Therefore, future research should focus on long-term adjunctive antidepressant treatment for schizophrenia negative symptoms to better confirm the efficacy of antidepressants in treating negative symptoms in schizophrenia.

Currently, distinguishing between primary and secondary negative symptoms remains a complex task, underscoring the need for future studies to delve into this topic with finer granularity and a more comprehensive approach. This meta-analysis further validates the effectiveness of antidepressant medications in the adjunctive treatment of negative symptoms, providing valuable insights for future research in this field. The positive and negative symptoms, as well as cognitive impairments, are core features of schizophrenia. Negative symptoms may manifest as a lack of motivation and the absence of elements that constitute normal social and emotional responses, but these are also common in depression (Malla, 1995). Siris et al., 1988 found that half of the patients with post-psychotic depression also met the criteria for negative symptoms, suggesting a syndromic overlap between depression and negative symptoms. Anhedonia, emotional blunting, irritability, motivation, sociability, and loneliness appeared in both the negative and depressive domains (Romney & Candido, 2001; Sax et al., 1996; Whiteford et al., 1987). A review (Krynicki et al., 2018) indicated that, despite considerable heterogeneity in research findings, certain depressive features (such as low mood, suicidal thoughts, and pessimism) differ from negative symptoms of schizophrenia, which are characterized by aphasia and emotional flatness. For instance, (Liemburg et al., 2013; Messinger et al., 2011) found that while depressive mood and suicidal intent reflect the depressive domain, emotional blunting and aphasia belong to the ‘expressive deficit’ domain of negative symptoms. The contribution of depression to negative symptoms is a possibility, and improvement of depression through antidepressant medications can influence the change in negative symptoms. It is a continuing debate whether the improvement is secondary to the reduction of depressive symptoms (Rummel et al., 2005). All studies included in this meta-analysis used standardized tools to assess negative symptoms, and changes in scores after intervention were standardized. This is primarily aimed to address the core issues of negative symptoms. Although this meta-analysis screened for depressive symptoms and focused on negative symptoms, the final observed effect may reflect improvements in depressive symptoms that were mistakenly attributed to negative symptoms. This is due to factors such as the inconsistency of assessment scales for depressive and negative symptoms across studies, as well as the variability in evaluation standards among experimenters.

When evaluating efficacy, it is important to not rely only on changes in scale scores. Given the high prevalence and significant burden of schizophrenia, particularly with regard to negative and depressive symptoms (Bobes et al., 2010; Buckley et al., 2009; Conley et al., 2007; Fervaha et al., 2014; Hawton et al., 2005; Mäkinen et al., 2008; Olivares et al., 2013; Rosen et al., 2006; Sands & Harrow, 1999; Tapp et al., 2001), even if a small proportion of patients exhibit symptom improvement, it can still be deemed clinically meaningful, as we have estimated. Moreover, beyond the effectiveness of interventions and the quality of supporting evidence, the perspectives of patients should also be taken into account (Hawton et al., 2005).

### Limitations

In the studies included in this meta-analysis, we focused more on research that screened for depressive symptoms in the inclusion criteria, regardless of the scale used to assess depressive symptoms. This refinement led to resulting in a relatively low proportion of depression in the overall psychopathology. While acknowledging the novelty and significance of our study, it is imperative to take into account the following limitations. (i) The number of RCTs comparing antidepressants with placebo in augmenting baseline antipsychotic treatment is relatively small, which precludes a more detailed analysis of individual antipsychotic-antidepressant combination therapies. (ii) Many of the included studies are small scale, so our results may be influenced by small study effects, including exaggerated effect sizes (Cumpston et al., 2019). However, since the risk of bias assessment is acceptable, this overestimation is likely to be relatively small. Clearly, large randomized controlled trials with sufficient power would help provide more definitive evidence. (iii) The types of antidepressants included in this study were based on existing randomized controlled trials, and the variety of antidepressants is not comprehensive. Therefore, future research on the treatment of negative symptoms of schizophrenia should focus on currently confirmed antidepressant medications that are effective for negative symptoms and collect more reliable and extensive data for further investigation. (iv) The included studies used various scales to assess negative symptoms. Although we made efforts to standardize the results, potential errors were not fully eliminated. (v) The included studies could not clearly specify the types of antipsychotic medications used in the background treatment, making it impossible to conduct further analysis.

### Conclusions

To the best of our knowledge, this study represents the first investigation into the use of antidepressants for treating negative symptoms in schizophrenia. In the current meta-analysis, we observed that mirtazapine and duloxetine exhibited the highest efficacy in the treatment of negative symptoms in patients with schizophrenia among antidepressants. The negative symptoms in schizophrenia patients present a significant challenge in current treatment approaches for the disorder. Despite the numerous proposed treatments for negative symptoms, their efficacy remains uncertain. Among these, antidepressants have been the most commonly suggested; however, their clinical application remains relatively limited. This research introduces a novel perspective on utilizing antidepressants for addressing negative symptoms in schizophrenia, potentially aiding in the identification of optimal treatment strategies. In this meta-analysis, the articles we included used different scales for the primary outcome measure, so although we used standardized mean differences (SMD) to eliminate their impact on the results, there still exist differences in the comprehensiveness of the negative symptom assessment between the two scales. Compared to PANSS-N, SANS represents more items; thus, SANS may provide more interpretable factors for discovery (Blanchard & Cohen, 2006). In future research, we propose the adoption of consistent scales for the evaluation of related indicators.

## Supporting information

Li et al. supplementary material Li et al. supplementary material

## Supplementary material

The supplementary material for this article can be found at http://doi.org/10.1017/S0033291725101803.
