# Supplementary material for: Efficacy of adjunctive antidepressants in treating negative symptoms of schizophrenia: a systematic review and network meta-analysis
Source: Psychol Med. 2025 Oct 24;55:e317. doi: 10.1017/S0033291725101803 (PMC12558627; doi:10.1017/S0033291725101803)
Supplement: Li et al. supplementary material [file S0033291725101803sup001.docx]

**supplementary materials**

**Supplementary Table 1**. The common and distinct features of the three symptom domains.

| features | primary negative symptoms | secondary negative symptoms | symptoms of major depression |
| --- | --- | --- | --- |
| common | anhedonia, anergia and avolition | anhedonia, anergia and avolition | anhedonia, anergia and avolition |
| distinguishing | an enduring deficitstate within schizophrenia;  alogia and blunted affect | manifestations of depressive features or medication side effects , or the consequence of positive symptoms;  alogia and blunted affect | low mood, suicidal ideation, and pessimism |


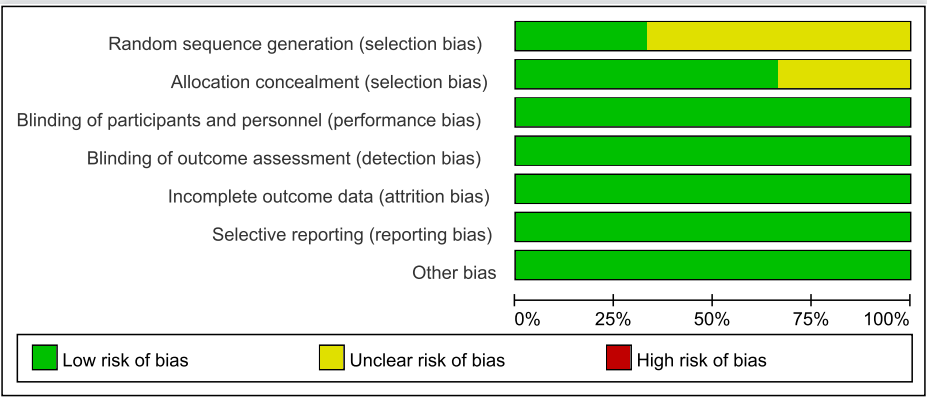


**Supplementary Figure 1.** Risk of bias summary.

**
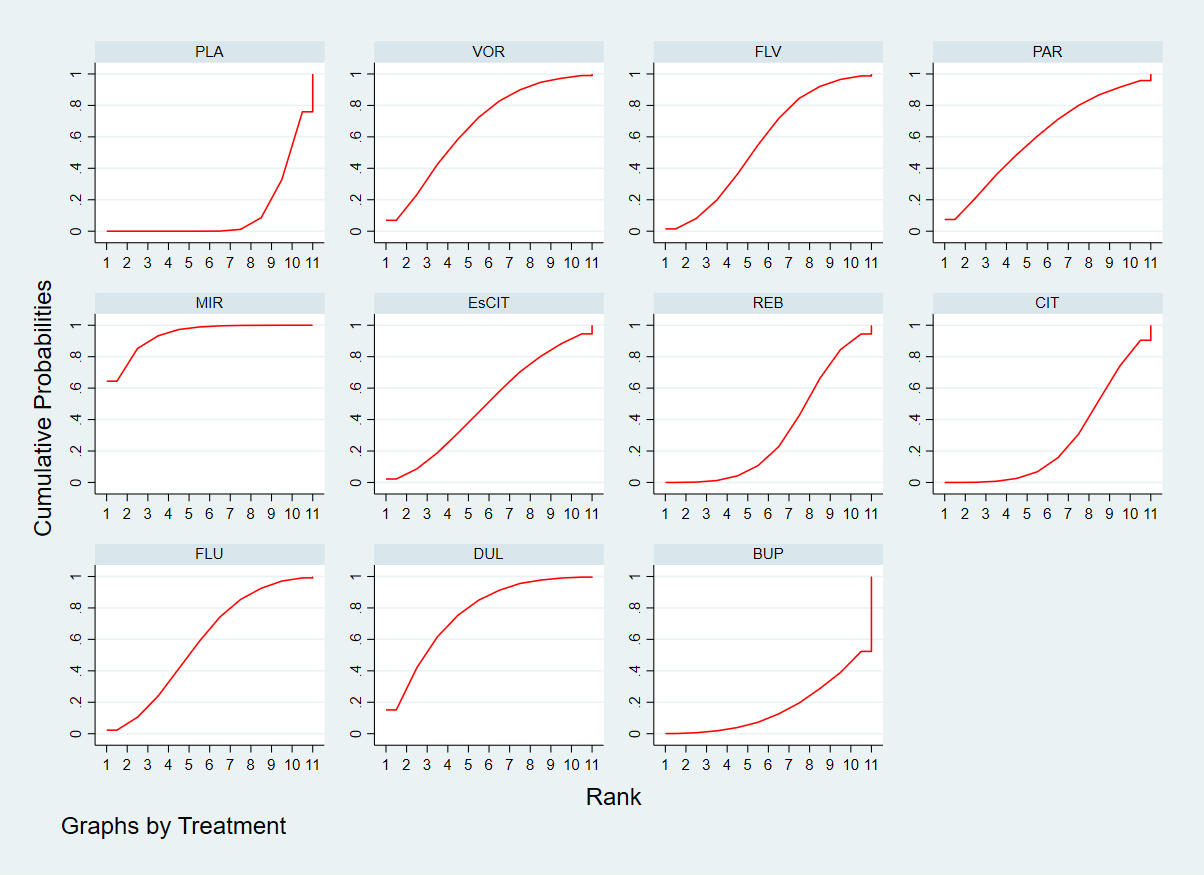
**

**Supplementary Figure 2.** The area under the curve plot. PLA represents placebo, VOR represents vortioxetine, FLV represents fluvoxamine,PAR represents paroxetine, MIR represents mirtazapine, EsCIT represents escitalopram, REB represents reboxetine, CIT represents citalopram, FLU represents fluoxetine, DUL represents duloxetine, and BUP represents bupropion.The greater the area under the curve for each drug, the better its efficacy.


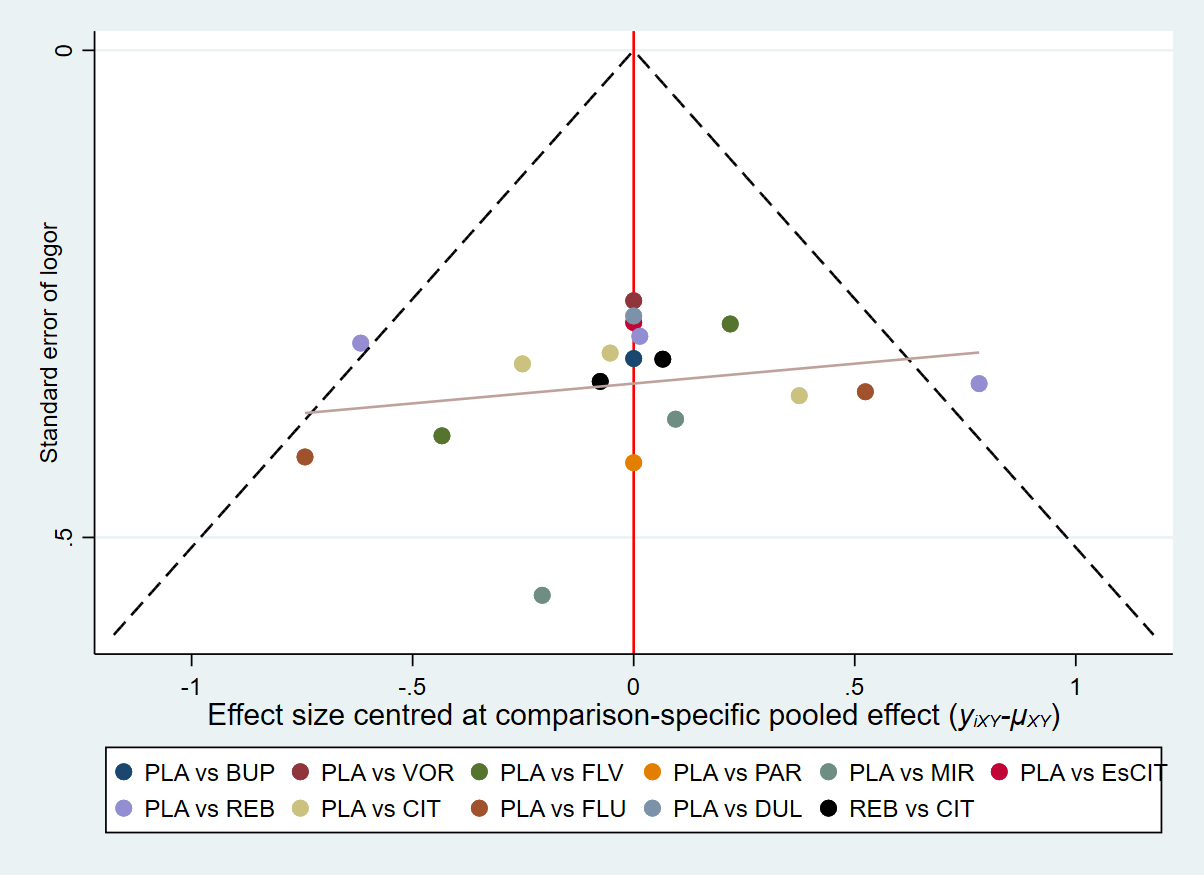


**Supplementary Figure 3.** Funnel plot. PLA represents placebo, VOR represents vortioxetine, FLV represents fluvoxamine,PAR represents paroxetine, MIR represents mirtazapine, EsCIT represents escitalopram, REB represents reboxetine, CIT represents citalopram, FLU represents fluoxetine, DUL represents duloxetine, and BUP represents bupropion.


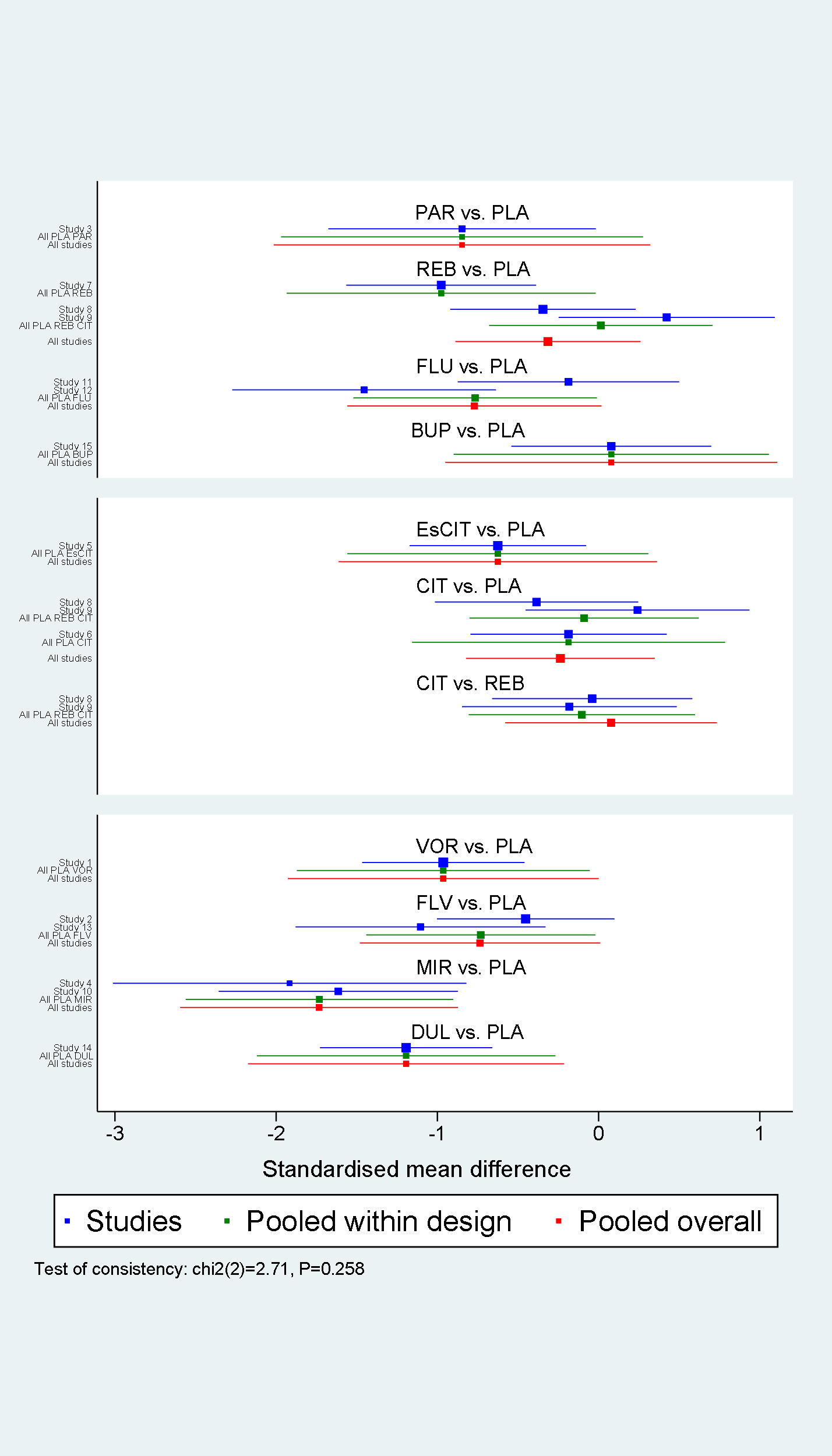


**Supplementary Figure 4.** Compare forest maps in pairs: PLA represents placebo, VOR represents vortioxetine, FLV represents fluvoxamine,PAR represents paroxetine, MIR represents mirtazapine, EsCIT represents escitalopram, REB represents reboxetine, CIT represents citalopram, FLU represents fluoxetine, DUL represents duloxetine, and BUP represents bupropion.


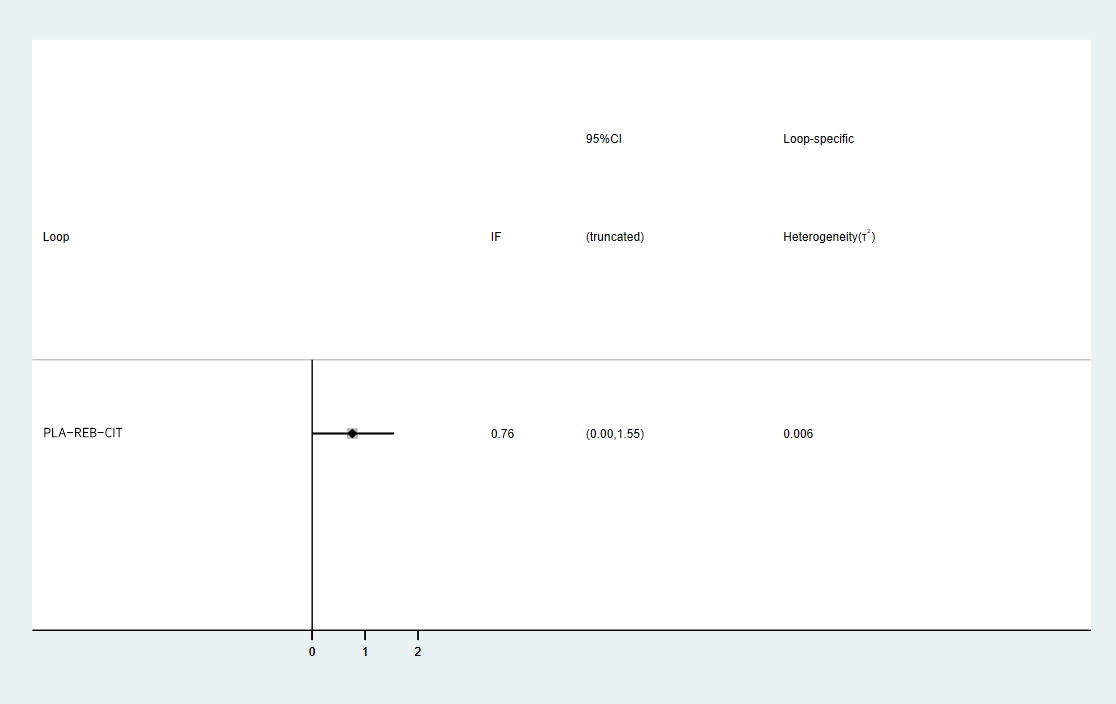


**Supplementary Figure 5.** Inconsistency Test. PLA: placebo; REB: reboxetine; CIT: citalopram.

**Search terms and strategies**

**Search terms**

Antidepressive Agents;Antidepressant Drug;Drug, Antidepressant;Antidepressants;Antidepressant;Antidepressant Drugs;Antidepressant Medication;Medication, Antidepressant;Antidepressive Agent;Agent, Antidepressive;Thymoanaleptics;Thymoanaleptic;Thymoleptics;Thymoleptic

Schizophrenia;Schizophrenias;Schizophrenic Disorders;Disorder, Schizophrenic;Disorders, Schizophrenic;Schizophrenic Disorder;Dementia Praecox

negative symptoms

**Pubmed**

("Schizophrenia"[MeSH Terms] OR "Schizophrenia"[All Fields] OR "schizophrenias"[All Fields] OR "schizophrenia s"[All Fields] OR ("Schizophrenia"[MeSH Terms] OR "Schizophrenia"[All Fields] OR ("schizophrenic"[All Fields] AND "disorders"[All Fields]) OR "schizophrenic disorders"[All Fields]) OR ("Schizophrenia"[MeSH Terms] OR "Schizophrenia"[All Fields] OR ("disorder"[All Fields] AND "schizophrenic"[All Fields]) OR "disorder schizophrenic"[All Fields]) OR ("Schizophrenia"[MeSH Terms] OR "Schizophrenia"[All Fields] OR ("disorders"[All Fields] AND "schizophrenic"[All Fields]) OR "disorders schizophrenic"[All Fields]) OR ("Schizophrenia"[MeSH Terms] OR "Schizophrenia"[All Fields] OR ("schizophrenic"[All Fields] AND "disorder"[All Fields]) OR "schizophrenic disorder"[All Fields]) OR ("Schizophrenia"[MeSH Terms] OR "Schizophrenia"[All Fields] OR ("dementia"[All Fields] AND "praecox"[All Fields]) OR "dementia praecox"[All Fields]) OR "Schizophrenia"[MeSH Terms]) AND ("Antidepressive Agents"[MeSH Terms] OR ("Antidepressive Agents"[Pharmacological Action] OR "Antidepressive Agents"[MeSH Terms] OR ("antidepressive"[All Fields] AND "agents"[All Fields]) OR "Antidepressive Agents"[All Fields] OR ("antidepressant"[All Fields] AND "drug"[All Fields]) OR "antidepressant drug"[All Fields] OR ("Antidepressive Agents"[Pharmacological Action] OR "Antidepressive Agents"[MeSH Terms] OR ("antidepressive"[All Fields] AND "agents"[All Fields]) OR "Antidepressive Agents"[All Fields] OR ("drug"[All Fields] AND "antidepressant"[All Fields]) OR "drug antidepressant"[All Fields]) OR ("antidepressent"[All Fields] OR "antidepression"[All Fields] OR "Antidepressive Agents"[Pharmacological Action] OR "Antidepressive Agents"[MeSH Terms] OR ("antidepressive"[All Fields] AND "agents"[All Fields]) OR "Antidepressive Agents"[All Fields] OR "antidepressant"[All Fields] OR "antidepressants"[All Fields] OR "antidepressive"[All Fields] OR "antidepressives"[All Fields]) OR ("antidepressent"[All Fields] OR "antidepression"[All Fields] OR "Antidepressive Agents"[Pharmacological Action] OR "Antidepressive Agents"[MeSH Terms] OR ("antidepressive"[All Fields] AND "agents"[All Fields]) OR "Antidepressive Agents"[All Fields] OR "antidepressant"[All Fields] OR "antidepressants"[All Fields] OR "antidepressive"[All Fields] OR "antidepressives"[All Fields]) OR ("Antidepressive Agents"[Pharmacological Action] OR "Antidepressive Agents"[MeSH Terms] OR ("antidepressive"[All Fields] AND "agents"[All Fields]) OR "Antidepressive Agents"[All Fields] OR ("antidepressant"[All Fields] AND "drugs"[All Fields]) OR "antidepressant drugs"[All Fields]) OR ("Antidepressive Agents"[Pharmacological Action] OR "Antidepressive Agents"[MeSH Terms] OR ("antidepressive"[All Fields] AND "agents"[All Fields]) OR "Antidepressive Agents"[All Fields] OR ("antidepressant"[All Fields] AND "medication"[All Fields]) OR "antidepressant medication"[All Fields]) OR ("Antidepressive Agents"[Pharmacological Action] OR "Antidepressive Agents"[MeSH Terms] OR ("antidepressive"[All Fields] AND "agents"[All Fields]) OR "Antidepressive Agents"[All Fields] OR ("medication"[All Fields] AND "antidepressant"[All Fields]) OR "medication antidepressant"[All Fields]) OR ("Antidepressive Agents"[Pharmacological Action] OR "Antidepressive Agents"[MeSH Terms] OR ("antidepressive"[All Fields] AND "agents"[All Fields]) OR "Antidepressive Agents"[All Fields] OR ("antidepressive"[All Fields] AND "agent"[All Fields]) OR "antidepressive agent"[All Fields]) OR ("Antidepressive Agents"[Pharmacological Action] OR "Antidepressive Agents"[MeSH Terms] OR ("antidepressive"[All Fields] AND "agents"[All Fields]) OR "Antidepressive Agents"[All Fields] OR ("agent"[All Fields] AND "antidepressive"[All Fields]) OR "agent antidepressive"[All Fields]) OR ("Antidepressive Agents"[Pharmacological Action] OR "Antidepressive Agents"[MeSH Terms] OR ("antidepressive"[All Fields] AND "agents"[All Fields]) OR "Antidepressive Agents"[All Fields] OR "thymoanaleptics"[All Fields]) OR ("Antidepressive Agents"[Pharmacological Action] OR "Antidepressive Agents"[MeSH Terms] OR ("antidepressive"[All Fields] AND "agents"[All Fields]) OR "Antidepressive Agents"[All Fields] OR "thymoanaleptic"[All Fields]) OR ("Antidepressive Agents"[Pharmacological Action] OR "Antidepressive Agents"[MeSH Terms] OR ("antidepressive"[All Fields] AND "agents"[All Fields]) OR "Antidepressive Agents"[All Fields] OR "thymoleptic"[All Fields] OR "thymoleptics"[All Fields]) OR ("Antidepressive Agents"[Pharmacological Action] OR "Antidepressive Agents"[MeSH Terms] OR ("antidepressive"[All Fields] AND "agents"[All Fields]) OR "Antidepressive Agents"[All Fields] OR "thymoleptic"[All Fields] OR "thymoleptics"[All Fields]))) AND (("negative"[All Fields] OR "negatively"[All Fields] OR "negatives"[All Fields] OR "negativities"[All Fields] OR "negativity"[All Fields]) AND ("diagnosis"[MeSH Subheading] OR "diagnosis"[All Fields] OR "symptoms"[All Fields] OR "diagnosis"[MeSH Terms] OR "symptom"[All Fields] OR "symptom s"[All Fields] OR "symptomes"[All Fields]))

**Web of Science**

<https://www.webofscience.com/wos/alldb/summary/54913a15-91e2-4849-a32b-49000b6710df-95d25a6f/relevance/1>

**Schizophrenia** (Topic) or **Schizophrenias** (Topic) or **Schizophrenic Disorders** (Topic) or **Disorder, Schizophrenic** (Topic) or **Disorders, Schizophrenic** (Topic) or **Schizophrenic Disorder** (Topic) or **Dementia Praecox** (Topic) and **Preprint Citation Index** (Exclude – Database) AND **Antidepressive Agents** (Topic) or **Antidepressant Drug** (Topic) or **Drug, Antidepressant** (Topic) or **Antidepressants** (Topic) or **Antidepressant** (Topic) or **Antidepressant Drugs** (Topic) or **Antidepressant Medication** (Topic) or **Medication, Antidepressant** (Topic) or **Antidepressive Agent** (Topic) or **Agent, Antidepressive** (Topic) or **Thymoanaleptics** (Topic) or **Thymoanaleptic** (Topic) or **Thymoleptics** (Topic) or **Thymoleptic** (Topic) and **Preprint Citation Index** (Exclude – Database) AND **negative symptoms** (Topic) and **Preprint Citation Index** (Exclude – Database)
